# Barriers and Facilitators to Home Dialysis Among Latinx Patients with Kidney Disease

**DOI:** 10.1001/jamanetworkopen.2023.28944

**Published:** 2023-08-15

**Authors:** Katherine Rizzolo, Rebeca Gonzalez Jauregui, Ileana Barrientos, Jade Teakell, Claudia Camacho, Michel Chonchol, Sushrut S. Waikar, Lilia Cervantes

**Affiliations:** 1 Section of Nephrology, Boston University Chobanian & Avedisian School of Medicine and Boston Medical Center, Boston, Massachusetts; 2 Anschutz Medical Campus, University of Colorado School of Medicine, Aurora; 3 Division of Renal Diseases and Hypertension, McGovern Medical School at UTHealth Houston, Houston, Texas; 4 Department of Medicine, University of Colorado-Anschutz Medical Campus, Aurora; 5 Division of Nephrology, University of Colorado-Anschutz Medical Campus, Aurora

## Abstract

**Importance:**

Latinx people have a high burden of kidney disease but are less likely to receive home dialysis compared to non-Latinx White people. The disparity in home dialysis therapy has not been completely explained by demographic, medical, or social factors.

**Objective:**

To understand the barriers and facilitators to home dialysis therapy experienced by Latinx individuals with kidney failure receiving home dialysis.

**Design, Setting, and Participants:**

This qualitative study used semistructured interviews with Latinx adults with kidney failure receiving home dialysis therapy in Denver, Colorado, and Houston, Texas, between November 2021 and March 2023. Patients were recruited from home dialysis clinics affiliated with academic medical centers. Of 39 individuals approached, 27 were included in the study. Interviews were audio-recorded, transcribed verbatim, and analyzed using thematic analysis.

**Main Outcomes and Measures:**

Themes and subthemes regarding barriers and facilitators to home dialysis therapy.

**Results:**

A total of 27 Latinx adults (17 [63%] female and 10 [37%] male) with kidney failure who were receiving home dialysis participated. Themes and subthemes were identified, 3 related to challenges with home dialysis and 2 related to facilitators. Challenges to home dialysis included misinformation and immigration-related barriers to care (including cultural stigma of dialysis, misinformation regarding chronic disease care, and lack of health insurance due to immigration status), limited dialysis education (including lack of predialysis care, no-nephrologist education, and shared decision-making), and maintenance of home dialysis (including equipment issues, lifestyle restrictions, and anxiety about complications). Facilitators to home dialysis included improved lifestyle (including convenience, autonomy, physical symptoms, and dietary flexibility) and support (including family involvement, relationships with staff, self-efficacy, and language concordance).

**Conclusions and Relevance:**

Latinx participants in this study who were receiving home dialysis received misinformation and limited education regarding home dialysis, yet were engaged in self-advocacy and reported strong family and clinic support. These findings may inform new strategies aimed at improving access to home dialysis education and uptake for Latinx individuals with kidney disease.

## Key Points

**Question:**

What are the barriers and facilitators experienced by Latinx individuals with kidney failure who initiate home dialysis treatment?

**Findings:**

In this qualitative study of Latinx people receiving home dialysis, barriers to home dialysis included misinformation, limited education, and issues with maintenance. Facilitators to home dialysis included an improved lifestyle and strong support system.

**Meaning:**

Addressing reported barriers and facilitators to home dialysis may help improve access to home dialysis for Latinx individuals with kidney disease.

## Teaser

This qualitative study evaluates the experiences of Latinx individuals with kidney failure who are receiving home dialysis.

## Introduction

A priority of the Advancing American Kidney Health Initiative, a 2019 presidential executive order, is to increase the rate of home dialysis use (peritoneal dialysis and home hemodialysis) over in-center dialysis for people with incident kidney failure in the United States. While Latinx individuals experience 2.1 times the incidence of kidney failure compared to non-Latinx White individuals,^1^ they are less likely to be treated with home dialysis.^1,2,3^ In the United States in 2020, 11.8% of Latinx individuals starting dialysis received dialysis at home compared with 14.7% of non-Latinx White individuals. After 1 year of dialysis, 16.5% of Latinx people receiving dialysis used home dialysis therapies, compared to 22.9% of non-Latinx White people receiving dialysis who used home therapies.^1^

Known facilitators to home dialysis uptake in non-Latinx populations include higher levels of education and socioeconomic status, predialysis care (allowing for adequate education and preparatory time for dialysis), and family and care support.^4,5,6^ Patient-level barriers described in non-Latinx populations include lack of social support, worry about performing independently, and inadequate space or housing.^5,7^ Worldwide, home dialysis use varies widely among Latin American countries^8^; Mexico has one of the highest rates of peritoneal dialysis use in the world. Despite this, the rate of home dialysis use among Latinx populations in the US is low, which may be due to difference in patient- and system-level factors in the US.^8,9,10^ However, home dialysis uptake by Latinx populations in the US is not completely understood even when adjusting for medical, demographic, and social factors.^2,3^ Thus, it is unclear why home dialysis use is lower in this group, as Latinx patient perspectives regarding barriers and facilitators to home dialysis in the US have not been adequately studied. Our objective was to understand the barriers and facilitators to home dialysis therapy decision-making, uptake, and maintenance through qualitative interviews with Latinx individuals with kidney failure receiving home dialysis.

## Methods

### Study Design

Semistructured one-on-one phone interviews were conducted between November 2021 and March 2023 in the participants’ preferred language (English or Spanish). Participants provided verbal consent to participate in the research study and received a $60 gift card as compensation. The study followed the Consolidated Criteria for Reporting Qualitative Research reporting guideline.^11^ The Colorado and Harris Health Multi-Institutional Review Board approved the study as exempt from ongoing review (category 2 exemption^12^).

### Setting and Participants

Eligible participants were English- or Spanish-speaking adults 18 years and older who were receiving home dialysis and who self-identified as Latinx/o/a and/or Hispanic. Participants were recruited from 3 home dialysis clinics affiliated with academic medical centers (2 in Denver, Colorado, and 1 in Houston, Texas). Initially, participants were recruited via convenience sampling of patients treated with home dialysis at 1 academic medical center in Denver. After initial analysis was completed, the Texas sample and second clinic in Denver were added in March 2022 using theoretical sampling (an iterative recruiting process where participants are recruited while data are being analyzed and theories are emerging).^13^ The invitation to participate occurred at their dialysis clinic appointment or by phone, and the semistructured interview and demographic questionnaire occurred via phone.

### Interview Guide

The interview guide was informed by an extensive literature review,^2,5,7,14,15,16,17,18,19^ which was used as sensitizing concepts^20,21^ to guide the direction of the initial interviews. Questions explored dialysis treatment decision-making, education, and health care navigation as well as transition to home dialysis (eAppendix in Supplement 1).

### Data Collection

Interviews were conducted by members of the study team (K.R., who is a nephrologist with prior qualitative research experience, and R.G.J., who is a Latina bilingual medical student trained in qualitative interviewing and methods by K.R., neither of whom had previous clinical interactions with participants, and C.C., who is a Latina bilingual patient navigator who had previous clinical interactions with some of the participants). Interviews were audio-recorded, transcribed verbatim, professionally translated to English, deidentified, and continued until thematic saturation (ie, when further observation and analysis do not reveal new concepts).^13,21,22^

### Analysis

Interview transcripts were analyzed contemporaneously by 3 members (K.R., R.G.J., C.C.) using atlas.ti version 9.22 (Atlas.ti Scientific Software Development) between November 2021 and March 2023. Coding and analysis were performed to inductively identify concepts using principles of thematic analysis.^23^ These concepts were grouped into initial themes and subthemes and then developed and refined the coding until they captured the participants’ perspectives of the barriers and facilitators to their home dialysis experience. A theoretical framework was developed through process of analysis and comparison of concepts^13^ using principles of grounded theory.^24,25,26^ Consensus on the framework was reached after review by study team members to ensure that the findings reflected the full range and depth of the data. Member-checking (returning the data to participants for accuracy) was not conducted.

## Results

Of the 34 people approached, 27 participants (79%; 17 [63%] female and 10 [37%] male) were interviewed (Table 1). Nonparticipation was due to lack of interest (n = 4), recent kidney transplant (n = 1), patient illness (n = 1), and lack of decision-making capacity per spouse (n = 1). Nineteen participants (70%) were born in Mexico; 22 (81%) were treated with peritoneal dialysis, and 14 (51%) received in-center hemodialysis prior to home dialysis. Twenty (74%) interviews were conducted in Spanish and the mean interview duration was 40 minutes. We identified 5 themes, 3 focused on barriers and 2 focused on facilitators (Table 2). A thematic schema was developed to illustrate associations among the themes (Figure).

**Table 1.** Participant Characteristics

Characteristic	No. (%) (N = 27)
Age range, y	
20-30	1 (4)
31-40	6 (22)
41-50	7 (26)
51-60	11 (41)
≥61	2 (7)
Sex	
Female	17 (63)
Male	10 (37)
Modality type	
Peritoneal dialysis	22 (81)
Home hemodialysis	5 (19)
Preferred language Spanish	20 (24)
Country of origin	
Mexico	19 (70)
United States	4 (15)
Other^a^	4 (15)
Self-reported limited English proficiency	16 (60)
Married or has a partner	12 (44)
Has children younger than 18 y	15 (56)
>3 People living in home	20 (74)
<High school education (n = 25)^b^	6 (22)
Working (full or part time)	10 (37)
Total years receiving dialysis, mean (SD)	4.1 (5.9)
Prior history of in-center hemodialysis	15 (56)
Prior history of kidney transplant	5 (19)
Waitlisted for kidney transplant (n = 23)^c^	6 (26)
Access to kidney physician before starting dialysis	16 (59)
Proximity to home dialysis clinic, mean (SD), min (n = 25)^d^	24 (13.2)
Self-reported history of diabetes	10 (37)

^a^ Including Puerto Rico, El Salvador, Honduras, and Guatemala.

^b^ Two patients reported unsure.

^c^ Four patients reported unsure.

^d^ Two patients reported unsure.

**Table 2.** Qualitative Themes, Subthemes, and Illustrative Quotes

Themes and subthemes	Quotes (participant ID number)
**Home dialysis misinformation and immigration-related barriers to care**	
Cultural stigma of dialysis	“Sometimes they stare at me. About a month ago I went to Mexico for two days to visit my relatives, and automatically, as soon as you arrive, it’s nice, but it’s sad. The first thing they say to you: ‘Oh, *how are you, keep fighting*’ and so on. But I’m fine, I’m making an effort. I mean, they think that because I’m on dialysis, I’m already ah, I’m already sad, I’m dying, this and that.” (22) “People make fun of dialysis people. And they make stupid jokes about, you shouldn’t live because you have a dialysis. You’re not normal like us.” (20) “Over [in Mexico], since there are no machines, like the ones they have over here, the ones for home dialysis like mine, yes. Because over there, unfortunately with the machines, power isn’t…it’s not safe, there are power outages all the time.” (22) “There is a lot of ignorance in the Hispanic community about this dialysis thing, I talked to my neighbor, and another friend I have, who were already on dialysis before me and are Hispanic. They are very afraid of getting infected.” (16)
Misinformation regarding chronic disease care	“The truth is that we Latinos are very close-minded. We are too close-minded. So, when we get to that point, the Latinos don’t know anything. So, their world collapses, they think they will draw their blood and who knows what they will do to them. So, as we are not informed, we find it difficult.” (11) “We simply need courses or someone to explain it to us, even if we don’t have relatives, but there is education, so that everyone can support us.” (22) “I think that some of the problems with the Hispanic people and dialysis would be the lack of knowledge about dialysis…before I had a problem with my kidney, I never even knew what dialysis was. That’s just not something that had ever crossed my path.” (21) “I identify as a Mexican American. I actually do have connections with my community and my culture. And I think that there’s a lot of unknowns for our community. I think people who do get on dialysis really don’t know a lot about it…a lot of my community is not as educated as I am. And so, they might need a lot of more detailed oriented kind of guidelines on how to really understand what it means the impact that it has on our life.” (27)
Lack of health insurance due to immigration status	“I have insurance now. The only bad thing is that when I went into the hospital the first time I didn’t have insurance and I’m paying an expensive bill.” (18) “The billing part of it has been difficult for me. I do work, so I have insurance through my work and then through my job and then I also have Medicare. So, getting those two, my [private insurance] and Medicare to coordinate in order to get my bills paid, it’s been difficult.” (21) “I had difficulty when I started with dialysis. I was swamped with debt with the hospitals…the state doesn’t give you anything.” (11)
**Limited dialysis education**	
Lack of predialysis care	“I thought it was going to be something like when you get sick, you take medicine until you get better and that’s it, but this is not like that. It’s something that I have to get used to. And they helped me with that. To understand that it’s not to get better.” (25) “I just went to the doctor and they told me that my kidneys were not working.” (7) “I found out about dialysis until I got sick, because since you’re not going through the situation, you’re not interested, until this happens to you.” (19) “Back in Mexico the doctor suddenly told me, ‘Your kidneys are working at 70%.’ I neglected myself because I didn’t feel bad, but suddenly I started to swell and I felt worried. I went to get checked and they were pretty damaged.” (9)
Nonnephrologist education	“My dad was also in dialysis. Automatically, they put him in home dialysis, so I saw that it was very convenient. I say, well, dad’s 80 and he can do it. Why can’t I? That’s when I decided to look into home dialysis.” (27) “I found about NxStage home dialysis on my own…nobody, my renal doctor, my main doctor, nobody knew about NxStage. I found NxStage in the internet and nobody knew the NxStage.” (23) “The doctor who told me about this wasn’t a specialist, it was my primary care doctor.” (14) “They told me to go to a dialysis talk…they explained to me what the dialysis was about. And the connection you had to dialysis.” (7)
Shared decision-making	“They didn’t give me any [dialysis education]. I was one of the ones that had a very bad experience. They just sent me into a center, sent me up into a center, my doctor, my renal doctor and that’s it.” (23) “When I started dialysis, I was going to [another hospital], and I guess they didn’t give me an option because they didn’t know anything about peritoneal dialysis back then.” (27) “The education at all was so minimal. The only option there was the blood one.” (20) “It was decided (by the doctors) because option they told me there was no room to do hemodialysis. There was no other option.” (2)
**Maintenance of home dialysis**	
Equipment issues	“It’s a hassle to travel with a machine to Mexico, that’s why I never took it again after two tries. I feel kind of stuck over here and pretty much all the family is there.” (23) “[My wife] was worried about the poor hygiene of my job. Because I am moving earth, rocks, things like that.” (15) “You have to have a specific place so that you have everything, all your supplies they give you. It has to be…a cool place, and I was like, my God, and now what? I have a room in the back, that room was emptied and we used it to store all that, and that was one of the things that worried me, because I said: And where am I going to put all that? And my room was far from the bathroom, so I had to switch rooms with one of my children and he gave me the room that is in front of the bathroom, because the machine must be connected to the bathroom drain.” (19) “[My daughter] does it for me, because I can’t see, I can’t see how I can get connected there.” (3)
Lifestyle restrictions	“I spend all my time doing it, because I do it four times a day, but I think I don’t qualify for the machine, according to what the doctor told me.” (6) “Dialysis at home requires cleaning your room, washing your bathroom, having your personal hygiene, eating very well. There are several things where your family or the people you live with have to help you.” (16) “Because I carry fluid every day in my belly, it’s—I’m not allowed to be picking up heavy stuff. It does change your lifestyle…there’s restriction. You need to take care of yourself.” (27) “The bad thing about peritoneal is you need to do it every day and it takes too many hours. It’s ten hours a day that I’m hooked up to the machine.” (2)
Anxiety about complications	“So, it was really hard for me…I was like, ‘I’m not going to be able to, I’m not going to be able to.’ But then, I said, ‘I have to be able to.’”(12) “Whenever I started doing it, I got very nervous, I mean, nervous, nervous, nervous. And I was starting to do it and I calmed down. And now I feel normal. In other words, I haven’t been nervous, this is easy for me.” (1) “And the concerns were infections. It was very easy to get an infection if you didn’t do it well or if you didn’t take care of yourself as they said.” (15) “[I fear that] something might happen…I think that’s the…the first thing I’ve heard. What if something happens, or I don’t have anyone to help me.” (14)
**Improved lifestyle**	
Convenience	“The advantage for me, really, is that I don’t leave my house. I don’t have to rush every day because I have to go to the clinic.” (12) “With the other dialysis that they offered me as well, the option they mentioned of going to the clinic to do the dialysis, well, you are kind of tied to them all the time.” (15) “I think there are just advantages of being able to do it at home. You get home from work, you take a shower, you eat, and then you connect yourself to the machine. You can watch TV. Go to the bathroom. You can do your stuff in your house. After you finish, you go to bed. The next day you wake up in the morning and the machine is done, you disconnect and go to work.” (15)
Autonomy	“I can go to the bathroom, I can sit down and watch TV, I can talk to my friends. It’s very nice. I don’t have to go to the hospital and stay there.” (20) “The house is 100% better, because in the house I had about ten hours to myself. I worked, I traveled, I ate, I drank water, I enjoyed life.” (16) “Just being able to be myself and be able to continue my lifestyle as I currently live it. I didn’t want any restrictions to inhibit my ability to, you know, really work on my health really work on my being available to my husband and my two children. You know, just not losing myself if that makes sense.” (26) “One of the biggest advantages is that I have more time with my family at home. Because when I used to go to the clinic, there were times when I had to leave work earlier because I was far away.” (17) “Because my whole family is in Mexico, it’s better for me to travel, that is, I take the things I need for the days I’m going to be there, and with hemodialysis, I have to go to a center and that is more difficult for me.” (4) “[In-center] was more restrictive in my perspective, because then I would be going into the office three times a week. And that would inhibit my ability to actually have a full-time job and be able to attend to my job duties, as well as attend to my health issues. So, when I looked at peritoneal, that just seems the most logical option for me.” (26) “I have more options to work longer. Because when I used to go to the clinic I wasted a lot of time.” (18) “The convenience of being able to keep working was good. To have a little more normal life.” (8)
Physical symptoms	“When I was going to the clinic for dialysis, I would suddenly get out of there dizzy and with the dialysis at home I woke up normal.” (1) “With hemodialysis. I felt that it left me without strength, that it left me with nothing. I mean, I left wanting nothing, nothing, nothing, nothing, I was very tired. And with the [peritoneal dialysis] one…I almost do everything as I normally would. I feel good, I feel rested, I feel comfortable. The only thing is that I have to be connected at night, but [peritoneal dialysis] is a thousand times better.” (22) “When I was going to the clinic, sometimes I was very tired from dialysis and the next day I had to work. Sometimes I didn’t feel like it. Now with home dialysis, I don’t have that.” (17)
Dietary flexibility	“I ate more strictly before, and when I started [home] dialysis, I was able to eat more things that I couldn’t eat before…it was forbidden for me to eat bananas, mangoes, avocadoes, everything that has a lot of potassium, and that is what I like the most.” (1) “Food, everything, that is potatoes, beans, guava. I mean, there are many of these things that I couldn’t eat, and now I can eat, but a little bit, not much.” (17) “Sometimes they still tell me that here, I should consume less potassium, fewer bananas, cheese, milk. The nutritionists tell me what to do, and I do what she says. Try not to eat so much cheese, milk, yogurt, or tortilla.” (13)
**Support**	
Family involvement	“I went there by myself and well when I got home I explained it to them and we shared thoughts. And so, we decided that it, the peritoneal method, well, was the best option.” (4) “They went with me, my daughter and my granddaughter. That way if I didn’t understand something we can talk about it. And we did talk about it right there and they agreed with my decision they said they were a good decision the one I choose.” (25) “My mom, she’s the one that helps me with, once I prep the machine, get everything going, then it’s time for me to connect the machine, she’s my person that helps me with that process.” (21) “I’ve had my daughter’s support. If you don’t have this support, it’s also very difficult.” (14)
Relationships with staff	“They are great. I have never been left with a question about anything. They answer everything. When I started, the doctor was very nice and told me how to get clean. The nurse, the social worker, the nutritionist looks for things to eat.” (8) “I was thinking that the support they give you is really helpful. Where they tell you, ‘Okay, I support you in this, so that you can achieve it.’” (12) “It’s like they become part of your family. You arrive and they treat you as if they had known you all your life, and they share things with you that—as if they were a part of your family. You can – they listen to you; they’re always attentive. For example, every time you go to the doctor, the social worker sees you, the nutritionist sees you, the nurse sees you, the doctor sees you. And if you need to talk to someone, you just tell them I need to talk to someone and that person shows up there in the office, and you can ask the questions you need to ask.” (19) “The experience is very good, whenever I talk to them, they always get back to me, whatever I need, they are always there.” (18)
Self-efficacy	“When I contracted the disease, I didn’t want my life to be over. So, I was obviously in a mindset to fight. I’ve always had that attitude in most of my life, because I’ve experienced being told no more than yes or obstacles to overcome rather than just the nonchalant response.” (24) “I had to research it and ask for it and tell her (the social worker) … and she said ‘Nope. Nope, you can’t. There’s no way, there’s no state that’s gone accept you like this, so you pretty much stuck here you can’t, go nowhere’ and she closed the door, yeah. I had to do everything myself.” (23) “I did all by myself. I did research. It took me days to find out that I love that dialysis. That it wasn’t going to be painful. And I saw all kinds of doctors doing it. So, I said, I want to be in it.” (20) “The social worker, who found this for me, she gave me all the information and I investigated it, because I am a person who investigates everything.” (19)
Language concordance	“The machine that I had, you press a button and it tells you step by step what to do, and it’s in Spanish.” (17) “I have a book that shows you step by step what to do…I have them in Spanish, they are like guides.” (1) “They always have a translator, and it’s very nice that way.” (9)

**Figure.**
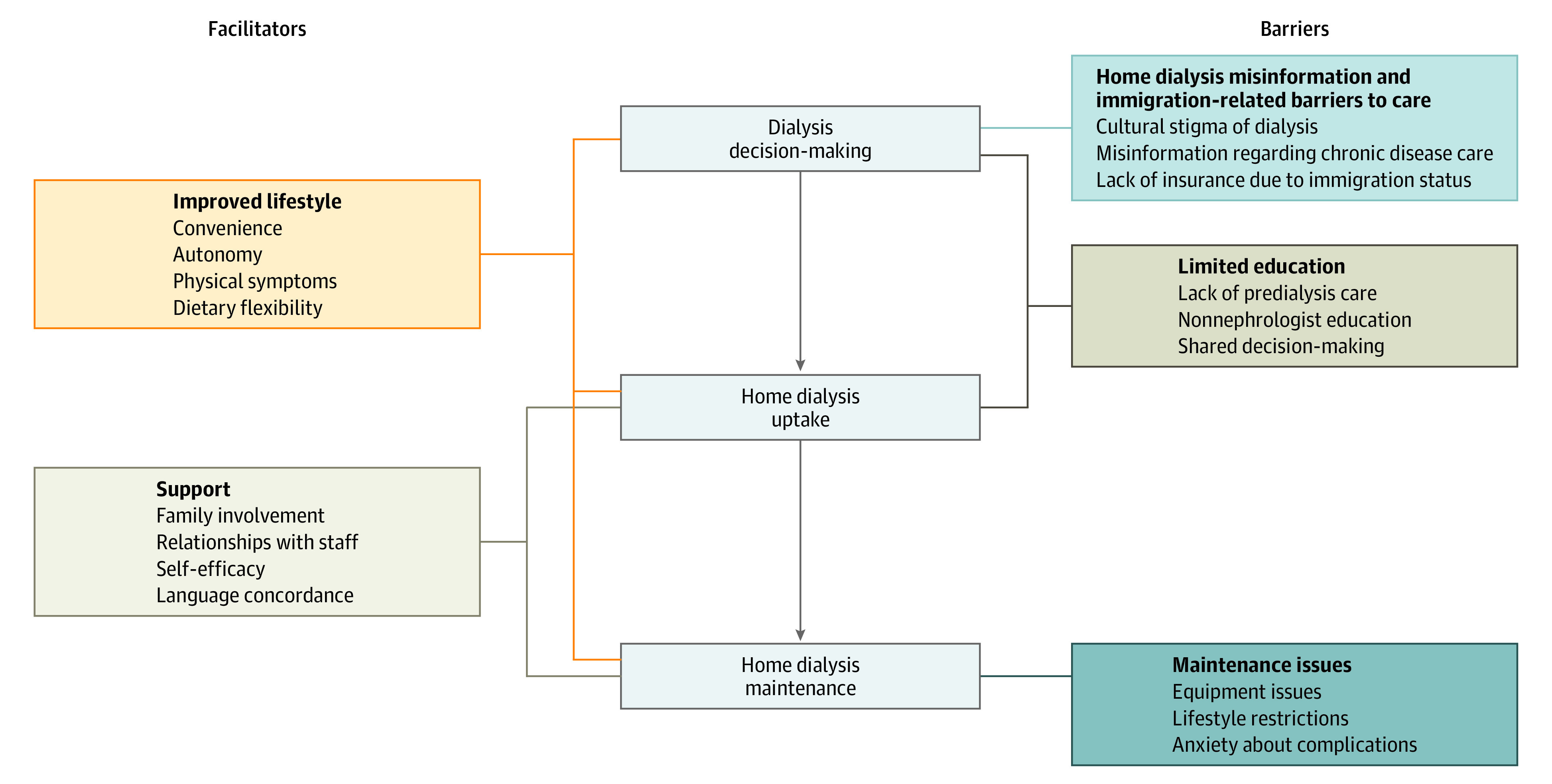
Thematic Schema Illustrating Associations Between Potential Barriers and Facilitators to Home Dialysis Decision-Making, Uptake, and Maintenance

### Home Dialysis Misinformation and Immigration-Related Barriers to Care

#### Cultural Stigma of Dialysis

Participants described a perception in the Latinx community that people receiving home dialysis are sick, which made participants feel stigmatized. One participant elaborated, “I remember that they used to say, that so-and-so have dialysis, automatically one would say he’s going to die, he’s too sick” (Participant 22). In addition, participants described perceptions of home dialysis being unsafe or dangerous compared with in-center dialysis in their country of origin. One participant, when asked what his family in Mexico thinks about home dialysis, remarked “Nobody would want me to be doing it” (Participant 2).

#### Misinformation Regarding Chronic Disease Care

Participants felt much of the cultural stigma regarding dialysis was due to lack of information in the community regarding kidney disease and need for regular medical care. One participant elaborated, “It’s just that us Latinos, those of us who speak Spanish, we don’t go to see the doctor. We go to the doctor when we are already sick” (Participant 11).

#### Lack of Health Insurance Due to Immigration Status

Undocumented participants reported that lack of health care insurance led to uncertainty about dialysis treatment options. One participant commented, “For a Latino, the most worrying thing is not having certainty. When I went to the hospital, I didn’t have insurance because I was illegal…in the first hospital, the doctor told me they couldn’t treat me here in the United States because I am illegal” (Participant 15).

### Limited Dialysis Education

#### Lack of Predialysis Care

Participants reported finding out about their kidney disease at a late stage or when they presented with symptoms. Others reported being told early on about their kidney disease but not understanding the implications of kidney disease progression; they did not know this was an end-stage, progressive disease. Others reported being told about their kidney disease by their primary care doctor but were not referred to specialty care until they started dialysis.

#### Nonnephrologist Education

Participants reported their nephrologist was not the main mechanism by which they heard about home dialysis. Participants described a range of ways in which they learned about home modality options, including friends, the internet, dialysis social workers and technicians, experience with family members who received home dialysis, and primary care physicians.

#### Shared Decision-Making

Many participants who received nephrology care before home dialysis (either predialysis or hemodialysis) reported that home dialysis was not discussed with them as an option. One participant said, “It wasn’t a great experience at the beginning and nobody offered nothing other ways, types, not even [peritoneal dialysis], nothing. They just put [you] into a center and you know how it is” (Participant 23).

### Maintenance of Home Dialysis

#### Equipment Issues

Participants described navigating issues with the peritoneal dialysis catheter and dialysis machine, and maintaining hygiene, such as infections and machine malfunctions. Concerns with space or physical limitations were also mentioned.

#### Lifestyle Restrictions

Participants reported multiple ways in which home dialysis affected their day to day life, including hygiene, frequency of treatments, home dialysis affecting other medical issues, and maintaining body image. One participant described how the weight restrictions in peritoneal dialysis affected their inability to work: “I can’t carry heavy things. I mean, my job, I used to work in construction” (Participant 22).

#### Anxiety About Complications

Participants recalled feeling anxiety and stress with home dialysis. Some of the concerns mentioned were fear of a bad outcome at home, fear of an infection, or worry that they would not be able to perform dialysis independently.

### Improved Lifestyle

#### Convenience

The convenience and flexibility of doing dialysis on their own schedules was a major motivator for participants. In particular, participants appreciated that they could modify their home dialysis schedules. One participant noted, “There’s flexibility to change my days, this is very difficult at the center, you have your schedule over there, a fixed schedule and you have to manage” (Participant 14).

#### Autonomy

Participants felt home dialysis offered a degree of control and autonomy over their treatments. Participants felt that administering their own treatment made them feel in control of their own disease. As one participant noted, “[When] I start putting my needles in, I start doing my treatments alone. I connected and disconnected myself. So, that gave me a lot of pride and independence” (Participant 23). Participants also felt that an advantage of home dialysis was more time with family. One participant said they opted for home dialysis, as “I have more time here at home with my child” (Participant 17). Many participants reported they chose home dialysis as it would allow them to keep working and support their families. One participant commented, “How was I going to survive, I couldn’t stop working…I really like dialysis at home because I can work” (Participant 16).

#### Physical Symptoms

Participants said that their symptom burden from kidney disease improved with home dialysis. Those who previously received in-center hemodialysis mentioned less fatigue and fewer symptoms of hypotension with home dialysis treatment. Participants reported that past negative experiences with hemodialysis, such as fatigue, feeling cold and uncomfortable, and having issues with their vascular access or needles, motivated them to opt for home dialysis.

#### Dietary Flexibility

Participants were glad to have a less restrictive diet compared with the chronic kidney disease or in-center hemodialysis diet, which allowed for more traditional Latinx foods, some of which are high in potassium. Despite this, many described difficulty in restricting their portion sizes and dairy intake. As one participant noted, “You know how we Latinos are. We are not full with a small portion, and we want more and more…but anyway, we learn, because in a month I have barely eaten cream or cheese, and I really like my wife to make me green enchiladas, or mole enchiladas, and I put in a lot of onion and cream” (Participant 11).

### Support

#### Family Involvement

Participants noted their caregivers and families were involved by attending clinic visits to learn about treatment options. Most participants reported their family supported them in home dialysis either directly (et, setting up the machine) or indirectly (eg, driving them to appointments and providing childcare or emotional support). Many felt their family’s support was invaluable in their home dialysis treatment.

#### Relationships With Staff

Once referred to the home dialysis clinic, participants reported that their experience going through home dialysis training was helpful, and they felt supported and reassured by the home dialysis training team. Many described that going through this training gave them confidence and helped to ease their anxiety. Multiple participants described having close relationships with the home dialysis clinic staff. As one participant put it, “It feels like they’re my family in reality…they helped me out and everything” (Participant 20). Participants also felt supported by the clinic in navigating ancillary needs, such as dialysis supplies, food, and insurance.

#### Self-Efficacy

Participants described advocating for their own care to overcome barriers to home dialysis education and treatment. Some participants reported they did their own research to advocate for their choice modality. One participant described moving to a different state in order to be accepted into a home hemodialysis program. Another described advocating to be changed back to peritoneal dialysis after undergoing temporary hemodialysis treatment in a rehabilitation center. Many described the importance of having a resilient attitude in overcoming fear and complications with home dialysis, following instructions, and being adherent with the training and treatments.

#### Language Concordance

Spanish-speaking participants felt they received adequate language concordant information from the home dialysis clinic during training and home dialysis treatment. The written materials and guides were in Spanish, and some clinics had language concordant staff while others used video or phone interpreters.

## Discussion

To our knowledge, this is the first qualitative study to characterize the facilitators and barriers to home dialysis decision-making, uptake, and maintenance experienced by a group of Latinx people receiving home dialysis. Facilitators of home dialysis included family caregiver and staff support with dialysis, as well as motivations for an improved lifestyle, especially in terms of working and spending time with family. Major challenges to home dialysis for Latinx participants in our study were misinformation and stigma surrounding home dialysis within the Latinx community and poor education regarding modalities. Our findings corroborate known motivators for home dialysis in non-Latinx populations that have not been described in Latinx groups (eg, flexibility, autonomy, and avoidance of hemodialysis)^10,13,14,15,16^ and barriers to home dialysis (eg, inadequate modality education, lifestyle restrictions, and issues with health care navigation)^7,27,28^ but also identified barriers to home dialysis that are potentially more unique to this population, such as dialysis stigma, misinformation about chronic disease care, and immigration status creating barriers to education and access. Increasing the use of home dialysis is a national priority; yet the models under the Advancing American Kidney Health initiative do not address many underlying factors affecting home dialysis uptake, such as the effects of poor predialysis education, cultural misinformation, and clinician-patient trust. An understanding of the challenges faced by Latinx populations pursuing home dialysis is important to inform culturally tailored strategies to reduce disparities in this population.

Latinx participants in our study received information about home dialysis from nonnephrologists, such as their primary care doctor, emergency department, and other community sources. Participants also described misinformation in the Latinx community that led to not seeking care as well as limited shared decision-making with their clinicians with respect to dialysis modality. Lack of predialysis education by nephrologists is a major barrier to home dialysis, as it limits timely dialysis decision-making and home dialysis training and preparation.^29,30^ Peritoneal dialysis is more often chosen when patients have received adequate modality education,^29^ but Latinx groups are less likely to receive adequate education compared with non-Latinx White individuals.^2,31^ Culture-concordant shared decision-making approaches has been shown to be important for Latinx populations navigating decisions such as contraceptive use,^32^ diabetes care,^33^ breast cancer,^34^ and palliative care.^35^ It is possible that lack of culture-concordant shared dialysis decision-making, combined with misinformation and dialysis stigma within the Latinx community limiting care seeking and earlier education, may contribute to low home dialysis uptake in Latinx groups in the United States.

The central role of family in Latinx culture regarding social support and family-oriented health care decisions is important to consider for dialysis modality decision-making.^35^ Participants in our study reported their families supported their modality decision and helped them with dialysis either directly or indirectly, which were likely a facilitator to their success with home dialysis and may be missing for Latinx individuals who do not take up home dialysis. Lack of caregiver support is a commonly cited barrier for non-Latinx populations, observed with higher frequency in low income households.^5^ Many described working to support family and being with family as a motivator to home dialysis that is important to their Latinx identity. Further, our finding of Latinx diet flexibility as a facilitator to home dialysis uptake is important to maintain close family connections and cultural identities through meal sharing, an important characteristic of Latinx culture.^36,37^ These findings stress the importance of including Latinx families in home dialysis education, training, and treatment. Understanding the values motivating a patient’s dialysis modality decision-making may help frame the discussion regarding benefits of home dialysis that are unique to Latinx individuals’ cultural identity.

Participants in our study felt the home dialysis clinic staff supported and listened to them, and they noted that education and resources were supplied in their preferred language, which was important to maintenance of home dialysis treatment. In contrast, Latinx and Black people with kidney disease have reported mistrust and discrimination by their dialysis medical clinicians and system.^38,39,40^ The close and trusting relationships reported by participants in our study illustrate the Latinx cultural value of *personalismo*, “a value for interacting with persons with whom one has a warm, caring, and trusting personal relationship.”^41^ This likely played a role in successful maintenance of home dialysis therapy. Establishing a close relationship between dialysis staff and patients may be critical to establishing the trust necessary for Latinx people to feel confident and safe to perform home dialysis.

### Limitations

Our study has limitations. The transferability of these findings to other dialysis centers or ethnic groups is uncertain. The sample in Texas comprised primarily individuals with undocumented immigration status, which may have led to a difference in structural barriers described by the participants included in the study. Additionally, what is referred to as the Latinx ethnic group is highly heterogeneous. Most of the participants in our study were of Mexican descent, which limits transferability to other Latinx communities. Moreover, as the participants in this study were successful with home dialysis uptake and maintenance, we were not able to elicit perspectives of individuals who did not commence or continue with home dialysis or who faced structural barriers impairing uptake. Future research including Latinx people who are not receiving home dialysis is critical to understanding additional barriers to home dialysis in this population.

## Conclusions

Overall, the Latinx people receiving home dialysis in this study received limited predialysis education and misinformation regarding home dialysis and yet were self-advocates with strong family and clinic support, which may have facilitated success with their uptake and maintenance of home dialysis treatment. As such, efforts toward improving home dialysis disparities for Latinx groups must focus on early culture concordant modality education—especially those that build patient activation and self-advocacy while incorporating Latinx cultural values.

## Notes

Supplement 1. **eAppendix.** Participant interview guide

Supplement 2.
Data Sharing Statement
