# Error in Abstract and Table

**DOI:** 10.1001/jamanetworkopen.2023.35236

**Published:** 2023-09-13

The Original Investigation titled “Barriers and Facilitators to Home Dialysis Among Latinx Patients with Kidney Disease,”^1^ published on August 15, 2023, was corrected to fix 2 percentage errors reported in Table 1 and the number of participants approached reported in the abstract.

## References

[1] Rizzolo K, Gonzalez Jauregui R, Barrientos I, . Barriers and facilitators to home dialysis among Latinx patients with kidney disease. JAMA Netw Open. 2023;6(8):e2328944. doi:10.1001/jamanetworkopen.2023.28944 37581885 PMC10427944

