# Supplementary material for: Barriers and Facilitators to Home Dialysis Among Latinx Patients with Kidney Disease
Source: JAMA Netw Open. 2023 Aug 15;6(8):e2328944. doi: 10.1001/jamanetworkopen.2023.28944 (PMC10427944; doi:10.1001/jamanetworkopen.2023.28944)
Supplement: Supplement 1. — eAppendix. Participant interview guide [file jamanetwopen-e2328944-s001.pdf]

Supplemental Online Content

Rizzolo K, Gonzalez Jauregui R, Barrientos I, et al. Barriers and facilitators to home dialysis among Latinx patients with kidney disease. *JAMA Netw Open*. 2023;6(8):e2328944. doi:10.1001/jamanetworkopen.2023.28944

eAppendix. Participant interview guide

This supplemental material has been provided by the authors to give readers additional information about their work.

© 2023 Rizzolo K et al. *JAMA Network Open.*

eAppendix. Participant interview guide

1. Establish a trusting and personalized relationship with the participant so that the participant does not feel threatened by the interview.
2. *Explore the home dialysis experience*
    a. Can you describe your experience receiving home dialysis?
    b. What is the best aspect of home dialysis? What is the worst?
3. *Explore the participant's pre-dialysis experience*
    a. Was home dialysis discussed with you as an option before you started on dialysis?
    b. Did you receive education regarding dialysis modalities before starting dialysis?
4. *Explore the participant's motivations for doing home dialysis*:
    a. Why did you choose to do home dialysis?
    b. Can you describe some of the conversations that took place about the pros/cons of each dialysis modality?
    c. Was your family part of the conversation regarding modality choice?
    d. Did you feel you received all the information that allowed you to make that decision?
    e. What were your greatest motivations/concerns regarding home dialysis? Did your family have additional concerns about home dialysis?
5. *Explore any challenges to home dialysis*:
    a. What has been challenging about home dialysis?
    b. Does anyone help you with dialysis at home?
    c. Do you have enough supplies?
    d. Do you have any side effects from the dialysis?
    e. How do you feel about your dialysis schedule?
    f. Tell me about your diet on dialysis.
6. *Explore the participant's experience with home dialysis education and clinic:*
    a. How has your experience been working with the clinic?
    b. Tell me about your home dialysis education (when starting)
    c. Do you have any issues with expenses or insurance for home dialysis?
    d. Do you feel the doctors and nurses listen to your concerns?
7. *Explore the Latino community's concerns about home dialysis:*
    a. What do you think is most concerning about home dialysis for the Latino community from XXX *(replace XXX with country of origin)*?

© 2023 Rizzolo K et al. *JAMA Network Open.*
