# Supplementary material for: Barriers and Facilitators to Home Dialysis Among Latinx Patients with Kidney Disease
Source: JAMA Netw Open. 2023 Aug 15;6(8):e2328944. doi: 10.1001/jamanetworkopen.2023.28944 (PMC10427944; doi:10.1001/jamanetworkopen.2023.28944)
Supplement: Supplement 2. — Data Sharing Statement [file jamanetwopen-e2328944-s002.pdf]

Data Sharing Statement

Rizzolo. Barriers and Facilitators to Home Dialysis Among Latinx Patients with Kidney Disease. *JAMA Netw Open*. Published August 15, 2023. doi:10.1001/jamanetworkopen.2023.28944

Data
Data available: Yes
Data types: Deidentified participant data
How to access data: Data will be shared upon request by emailing katherine.rizzolo@cuanschutz.edu
When available: With publication

Supporting Documents
Document types: None

Additional Information
Who can access the data: to research whose proposed use of the data has been approved
Types of analyses: for a specified purpose
Mechanisms of data availability: after approval of a proposal
